# Report of Mortality in Buffalo for the Month of Feb., 1857

**Published:** 1857-04

**Authors:** C. L. Dayton

**Affiliations:** Health Physician


ART. IX.—*Report of Mortality in Buffalo for the month of Feb.*, 1857. By C. L. DAYTON, M. D., Health Physician.

| DISEASES. | No. | Males. | Females. |
|---|---|---|---|
| Accidens, Boiler Explosion, | 8 | 8 | |
| Apoplexia, | 1 | | 1 |
| Asphyxia Immersorum, | 1 | 1 | |
| Cancer of the stomach, | 1 | | 1 |
| " " breast, | 1 | | 1 |
| Croup, | 5 | 3 | 2 |
| Diarrhœa, | 1 | 1 | |
| Dysinteria, | 1 | | 1 |
| Debilitas Senilis, | 6 | 2 | 4 |
| Eclampsia, | 24 | 14 | 6 |
| " et Effusio Cerebri, | 1 | | 1 |
| Febris Typhoid, | 2 | | 2 |
| " Typhus, | 3 | 2 | 1 |
| " Scarlatina, | 39 | 17 | 21 |
| " " Maligna, | 2 | 1 | 1 |
| Gastritis Acuta, | 1 | | 1 |
| Hyperæmia Cerebralis, | 1 | | 1 |
| Hernia Inguinalis, | 1 | | 1 |
| Hydrops, | 3 | 1 | 2 |
| Hydrocephalus, | 6 | 3 | 3 |
| Ignotus, | 24 | 14 | 8 |
| Intemperance, | 1 | | 1 |
| Insanity, | 1 | | 1 |
| Icterus, | 1 | 1 | |
| Metritis, | 1 | | 1 |
| Marasmus, | 3 | | 3 |
| Morbus Encephalosis, | 1 | | 1 |
| Mortificatio Uteri, | 1 | | 1 |
| Phreno Pneumonitis, | 1 | 1 | |
| Peritonitis Puerperarum, | 1 | | 1 |
| Paralysis, | 1 | | 1 |
| " Pulmonalis, | 1 | 1 | |
| Poisoned by eating Maeches, | 1 | | 1 |
| Peritonitis, | 1 | | 1 |
| Pneumonitis, | 6 | 2 | 2 |
| Premature Birth, | 2 | | 2 |
| Rheumatism, Sub-acute, | 2 | 2 | |

REGISTER OF MORTALITY—CONTINUED.

| DISEASES. | No. | Males. | Females. |
|---|---|---|---|
| Syphilis Congenital, | 1 | | 1 |
| Splenitis, | 1 | 1 | |
| Scrophula, | 1 | | 1 |
| Supposed Infanticide, | 1 | | 1 |
| Suicide by taking opii, | 1 | 1 | |
| Still-born, | 9 | 4 | 3 |
| Tuberculosis Pulmonalis, | 20 | 7 | 13 |
| Total, | 191 | | |

SEXES.

| | |
|---|---|
| Males, | 87 |
| Females, | 93 |
| Sex not given, | 11 |
| Total, | 191 |

AGES.

| | | | |
|---|---|---|---|
| Still-born, | 9 | Between 10 years and 20 years, | 6 |
| 1 day, | 4 | " 20 " " 30 " | 24 |
| 1 day and 30 days, | 13 | " 30 " " 40 " | 16 |
| Between 1 month and 6 months, | 22 | " 40 " " 50 " | 9 |
| " 6 months and 12 " | 13 | " 50 " " 60 " | 5 |
| " 1 year " 3 years, | 27 | " 60 " " 70 " | 5 |
| " 3 " " 5 " | 19 | " 70 " " 80 " | 3 |
| " 5 " " 10 " | 12 | " 80 " " 90 " | 0 |
| | | " 90 " " 100 " | 0 |
| Total number under 10 years, | 119 | Total number over 10 years, | 68 |
| Ages not given, | 4 | Total, | 191 |

NATIVITIES.

| | | | |
|---|---|---|---|
| American, (white,) | 108 | Canadian, | 2 |
| " (colored,) | 1 | French, | 2 |
| German, | 26 | Scotch, | 0 |
| Irish, | 20 | Holland, | 3 |
| English, | 7 | Country not given, | 22 |
| Total, | | | 191 |

---

*Collodion for Sore Nipples.*—Dr. Simpson says, "in two cases I lately united the edges of the fissures, and covered them over with the solution of gun-cotton, making the layer pretty strong. It acted successfully, by maintaining the edges so firmly together that they were not again re-opened by the suction of the infant. The gun-cotton dressing is not like other dressings, affected by the moisture of the child's mouth; and, at the same time, by securing rest to the part, it allows complete adhesion and cicatrization speedily to take place. It is improved by the addition of a little olive oil."

